# Research on Fractal Characteristics and Energy Dissipation of Concrete Suffered Freeze-Thaw Cycle Action and Impact Loading

**DOI:** 10.3390/ma12162585

**Published:** 2019-08-14

**Authors:** Yan Li, Yue Zhai, Xuyang Liu, Wenbiao Liang

**Affiliations:** 1 School of Geology Engineering and Geomatics, Chang’an University, Xi’an 710054, China; 2 School of Civil Engineering, Chang’an University, Xi’an 710061, China

**Keywords:** concrete, freeze-thaw cycle action, SHPB, impact loading speed, fractal characteristic, energy dissipation

## Abstract

In order to study the fractal characteristics and energy dissipation of concrete suffered freeze-thaw cycle actions and impact loading, C35 concrete was taken as the research object in this paper, and freeze-thaw cycle tests were carried out with a freeze-thaw range of −20 °C~20 °C and a freeze-thaw frequency of 0~50 times. The degradation characteristics of concrete material and the variation rules of basic physical parameters under various freeze-thaw cycle conditions were obtained consequently. By using the SHPB (separated Hopkinson pressure bar) test device, impact compression tests of concrete specimens under different freeze-thaw cycle actions were developed, then the process of impact crushing and the mechanism of damage evolution were analyzed. Based on the screening statistical method and the fractal theory, the scale-mass distribution rules and fractal dimension characteristics of crushing blocks are investigated. Furthermore, the absorption energy, fracture energy and block kinetic energy of concrete under different conditions were calculated according to the energy dissipation principle of SHPB test. The relationship between the energy consumption density and the fractal dimension of fragments was established, and the coupling effect mechanism of freeze-thaw cycle action and strain rate effect on the fractal characteristics and energy consumption was revealed additionally. The research results show that the concrete under different freeze-thaw cycle conditions and impact loading speeds has fractal properties from the microscopic damage to the macroscopic fracture. The energy dissipation is intrinsically related to the fractal characteristics, and the energy consumption density increases with the increase of the fractal dimension under a certain freeze-thaw cycle condition. When at a certain loading speed, with the growth of freeze-thaw cycles, the energy consumption density reduces under the same fractal dimension, while the fractal dimension improves under the same energy consumption density.

## 1. Introduction

Concrete is the most widely used building material in the fields of national defense, civil defense and civil construction engineering [1]. In recent years, with the continuous expansion of the use of concrete (for example, nuclear reactor engineering, deep underground engineering, etc.), and the rapid development of various sophisticated strike weapons, many concrete structures are facing the threat of extreme external loads such as impact, explosion, etc. The cold regions of China are widely distributed, and the seasonal and permanent cold regions account for more than three quarters of China’s national territory. The cold area concrete projects will work for a long time in the freeze-thaw circulation environment. The remaining water in the interconnected narrow pores will continue to freeze and thaw, and will then cause the continuous development of internal pores, and thus the structural performance of concrete will be greatly affected. Therefore, studying the broken characteristics and energy dissipation of concrete under freeze-thaw cycle actions and impact loadings is of great theoretical value and practical significance to reasonably design the anti-impact of concrete protection projects in cold regions, and evaluate and reinforce the safety of post-disaster concrete structures.

The essence of the impact failure of materials is the energy consumption process, in which the internal damage cracks continue to expand and extend under the drive of external energy, eventually leading to material instability and failure [2,3]. The number, size and scale distribution rules of broken blocks after destruction is the macroscopic representation of this process [4]. The analysis of fractal characteristics and energy dissipation of concrete under freeze-thaw cycle conditions plays an important role in the research of the protection ability of concrete defense projects and the damage prediction of destroyed structures in cold regions. At present, the fractal theory is commonly used to study breaking laws of brittle materials such as concrete and rocks. It was established by Mandelbrot [5] in the 1970s and its research object is the disordered (irregular) self-similar system widely existing in nature. With the help of the fractal theory, we can study some intrinsic rules hidden behind complex phenomena. Studies have shown that [6,7,8,9,10], there are distributed at random a large number of mesoscopic damage structures inside the concrete, including pore spaces, microcracks, weak impurities and so on, whose distribution rules and geometrical shapes have significant statistical self-similarity within a certain range. These mesoscopic damage structures gestate, develop and converge continuously under the outer loads, thus the broken blocks of concrete have a certain fractal characteristics. However, most existing studies focus on the static or the dynamic performance of concrete materials at normal temperature or high temperature [4,11,12,13,14,15,16,17,18], and there are relatively few researches on mechanical properties of concrete under the coupled action of impact loading and freeze-thaw cycles, and the representative studies mainly include: Sun introduced the damage of concrete under the simultaneous action of load and freeze-thaw cycle and its dependence on concrete of different strength grades [19]; Xiao conducted separated Hopkinson pressure bar (SHPB) tests on four kinds of ceramsite concrete with different volume content at different freezing-thawing cycle condition, and obtained the empirical formula of relative dynamic maximum stress of ceramsite concrete [20]. Nevertheless, none of the above studies takes into account the impact fracture fractal and energy dissipation characteristics of concrete materials under the coupled action of freeze-thaw cycle and impact loading.

Since C35 concrete is commonly used in the concrete engineering in China, the impact compression test of C35 concrete suffered from different freeze-thaw cycles (0, 10, 20, 30, 40, 50), was conducted on the Φ 50 mm SHPB system in this paper. Through screening statistics of broken blocks under different conditions, the scale-mass distribution rules were gained. On the basis of the fractal theory and the energy dissipation principle of SHPB test, the fractal dimension and energy consumption characteristics of concrete fragments were analyzed, the relationship of energy consumption density and fractal dimension was established, and the influence mechanism of freeze-thaw cycle action and load speed was revealed.

## 2. Freeze-Thaw Cycle Test and Variation Rules of Basic Physical Parameters

### 2.1. Freeze-Thaw Cycle Test

C35 concrete was used in this paper, and its specific mixing ratios was calculated according to “the design rules of mixing ratio of ordinary concrete” (JGJ55-2011), as shown in Table 1.

The specific parameters and performance of the ingredients used to produce the mixture were shown as follows: (1) Cement. The cement was ordinary Portland cement with the strength grade of P.0.42.5 produced by Shaanxi Qinling Cement Building Materials Co., Ltd., Xi’an, China, and the physical and chemical properties were just as shown in Table 2. (2) Gravel. According to “Standard for technical requirements and test method of sand and crushed stone (or gravel) for ordinary concrete” (JGJ52-2006), gravel with continuous gradation of 5–16 mm was selected and screened with a sieve with sieve hole diameter of 2.75–19 mm. The gravel particle grading was shown in Table 3. (3) Sand. According to “Standard for technical requirements and test method of sand and crushed stone (or gravel) for ordinary concrete” (JGJ52-2006), continuous graded medium sand was selected, the fineness modulus of which was 2.42. The sand was used after washed and dried and the particle screening results was shown in Table 4. (4) Fly ash. Secondary fly ash with fineness of 43 μm was used with a density of 2.4 g/cm^3^ and moisture content of about 5%. (5) Water reducing agent. The PH value of the water reducing agent was 7–9 and the water reduction rate was about 20–35%. Prepared solutions with a concentration of 26–28% were used. (6) Water. Laboratory tap water, in compliance with standard requirements.

The well-mixed concrete materials were poured into the molds and maintained for 28 days in the standard curing room with a temperature of 20 °C and humidity of 95%. Through coring and cutting, the concrete was made into cylinders of Φ 48 mm × 25 mm. The upper and lower surfaces of the specimens were polished using a grinder during the specimen processing. The processing accuracy was strictly controlled to ensure the errors of both the surface planeness and the vertical deviation of upper and lower surfaces were within ±0.02 mm [21]. After specimens were numbered and their volumes and qualities were measured, they were placed in a drying box for 48 h. Then, they were moved into a saturated water dish, in which distilled water was injected and the air was extracted at pressure of 0.1 MPa for 4 h until no bubbles were spilled on the specimen surface. Then. they continued to soak for more than 24 h to obtain saturated concrete specimens. Following this, the mass of the saturated specimens was weighed, and the physical parameters including saturated density, saturated moisture content, porosity, etc. were calculated.

When developing the freeze-thaw cycle test, the saturated concrete specimens were firstly put into the automatic low-temperature freeze-thaw test box (Model: CLD-1. Temperature measurement accuracy: ±0.5 °C. Temperature range: −40~+50 °C), and then the freeze-thaw cycle tests were conducted with freeze-thaw temperature varying from −20 °C to +20 °C and freeze-thaw cycle numbers changing from 0 to 50 times. The process of per freeze-thaw cycle was as follows: at first, the temperature was slowly reduced from 20 °C to −20 °C (this process took 120 min), keeping a constant temperature for 240 min, then rapidly rising to room temperature of 20 °C (this process took 30 min), keeping constant temperature for 240 min. The total time of one freeze-thaw cycle is 630 min, and 10 freeze-thaw cycles are defined as a phase.

### 2.2. Freeze-Thaw Deterioration Characteristics of Concrete

**Figure 1:**
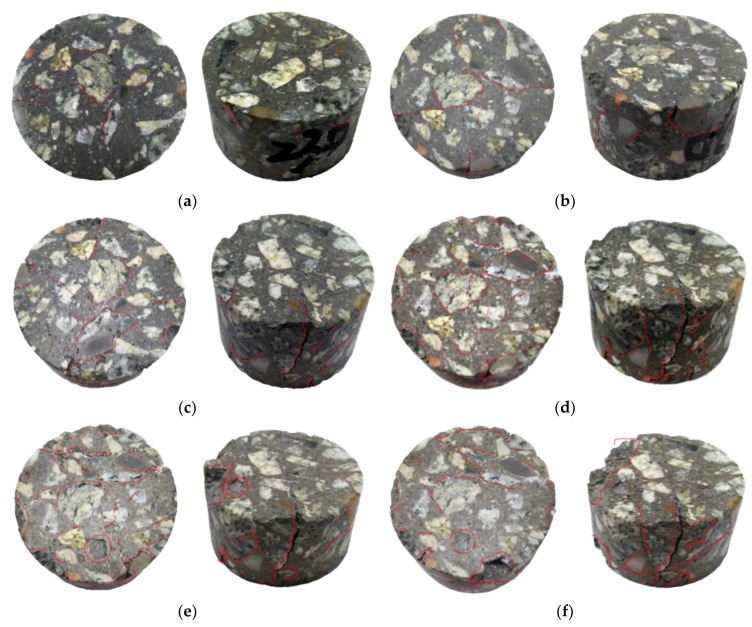
Deterioration characteristics of concrete specimens under freeze-thaw actions. (**a**) 10 freeze-thaw cycles; (**b**) 20 freeze-thaw cycles; (**c**) 30 freeze-thaw cycles; (**d**) 40 freeze-thaw cycles; (**e**) 50 freeze-thaw cycles; (**f**) 60 freeze-thaw cycles.

After different freeze-thaw cycles, the internal defects of concrete specimens propagated, expanded, gathered and penetrated continuously, and eventually developed from microscopic damage to macroscopic fracture. The apparent crack propagation and development of concrete specimens under different freeze-thaw conditions were shown in Figure 1.

Figure 1 showed that, after experiencing 10 freeze-thaw cycles, the surface deterioration characteristics of the concrete specimen were mainly reflected on the slight peeling off the edge of coarse aggregate and cementing materials, and “cracking phenomenon” began occurring on the specimen surface at this time. After suffering 20 freeze-thaw cycles, the cementing material fell off slightly at the edge of specimens, mainly concentrating near the coarse aggregate, and obvious cracks appeared at the interface between coarse aggregate and cementing material. After undergoing 30 freeze-thaw cycles, cracks on the surface of specimens developed continuously, and the number and width of cracks had increased. The aggregation of cracks led to a large crack along the axial direction of the specimen, accompanied by a small amount of spalling of edge aggregates and colloids. After 40 freeze-thaw cycles, the surface cracks of specimens increased and widened significantly, and the axial cracks further extended and penetrated, with new cracks generated along the aggregate-colloid interface. The growth and connection of multiple cracks increased the peeling area of the specimen edge. After being frozen and thawed 50 times, the main cracks of concrete specimens were connected, and the aggregate peeled seriously from the edges. The concrete specimen had begun to take on an incomplete form. When freeze-thaw cycles reached 60 times, several main cracks were connected through the specimen interior, the bulks of the specimen peeled off, and the appearance deteriorated seriously, and as a result, the specimen lost its integrity and could not perform the mechanical test. Therefore, the upper limit number of freeze-thaw cycles in the follow-up study of this paper was 50 times.

According to the above analysis of the apparent deterioration characteristics and damage evolution processes of concrete under the freeze-thaw cycle actions, the mechanism of freeze-thaw cycle deterioration of concrete is as follows: the concrete is a kind of non-homogeneous material and there exists internal microcracks and micro pores. The internal freeze-thaw damage of concrete specimens starts from the freezing and thawing of the water in internal initial pores of materials, and the external apparent degradation begins at the aggregate-colloid interface. As the cracks grow and extend continuously, the damage develops further to the spalling of aggregates, colloids, and even concrete blocks. This is because after the water inside the concrete freezes, the specimen volume expands and the frost heaving force causes continuous development of internal pores; the water in the pores further increases, then the specimen volume continues to expand, resulting in the more severe deterioration and the loss of integrity.

### 2.3. Variation Rules of Basic Physical Quantity

**Figure 2:**
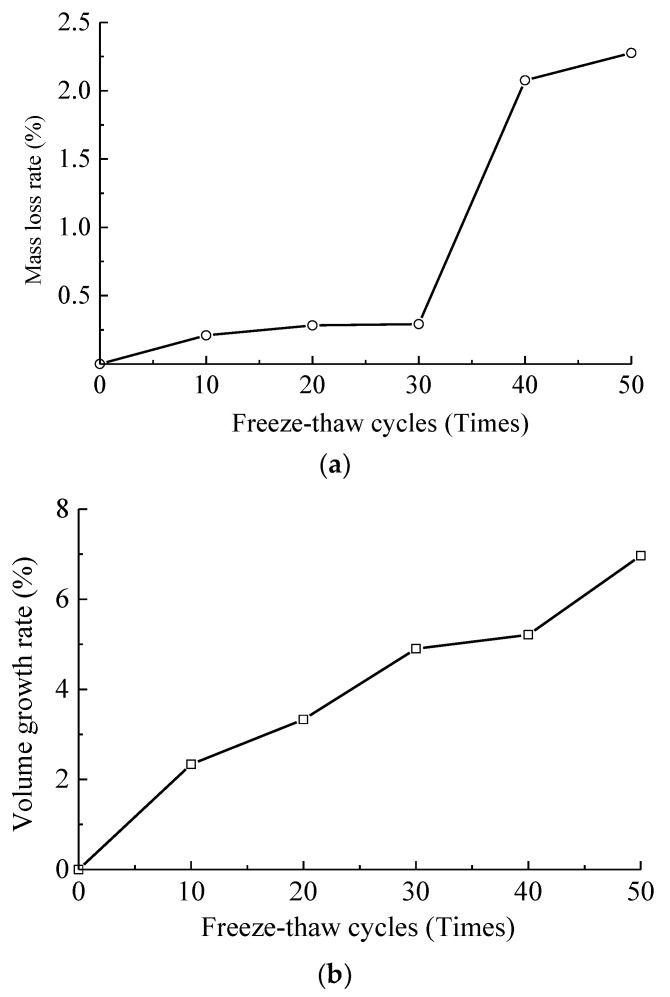

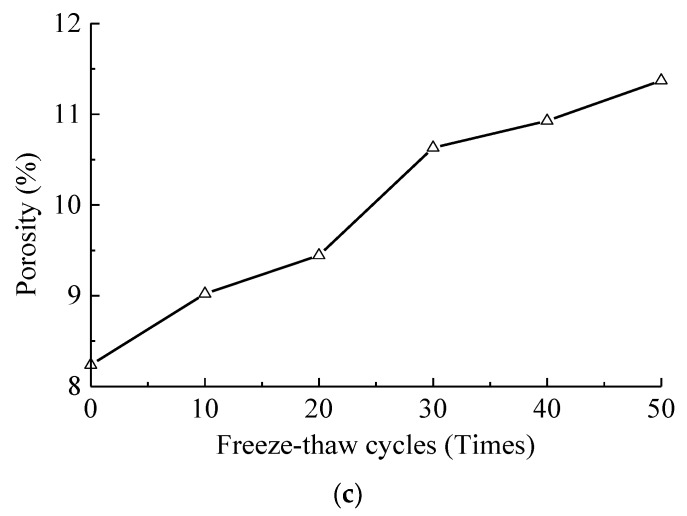
Variation rules of basic physical parameters of concrete specimens under freeze-thaw conditions. (**a**) Mass loss rate; (**b**) Volume growth rate; (**c**) Porosity.

In the freeze-thaw test, the mass and the volume of specimens were measured, and the porosity was calculated per 10 freeze-thaw cycles. Finally, the variation rules of the mass, volume and porosity of concrete under different freeze-thaw cycle actions were gained as shown in Figure 2.

As found in Figure 2a, since the spalling of aggregates, colloids and concrete blocks occurring in the freeze-thaw cycle test, the mass of specimens decreased, and the mass loss rate of specimens increased with the increase of freeze-thaw cycles consequentially. When the freeze-thaw cycles ranged from 10 to 30 times, the mass loss rates of specimens were 0.21%, 0.28% and 0.29%, respectively, and the mass loss rate changed slowly in this process. However, when it reached 40 freeze-thaw cycles, the mass loss rate was rapidly increased to 2.08%. This was because the concrete specimens began to produce a large spalling area of aggregates, colloids, even blocks at this moment, and the deterioration was more serious. While the freeze-thaw cycle number rose to 50 times, the mass loss rate was raised to 2.28%.

As indicated in Figure 2b,c, the volume and the porosity of concrete enlarged linearly with the increase of the freeze-thaw cycles. As the freeze-thaw cycles varied from 10 to 50, the volume growth rates enlarged from 2.34% to 3.33%, 4.90%, 5.21%, 6.97% respectively, and the porosity improved from 8.24% (the porosity without freeze-thaw cycle action, namely the initial porosity) to 9.02%, 9.44%, 10.63%, 10.93% and 11.37% respectively. During the freeze-thaw process, the alternating action of water and ice makes the pores inside the concrete increase continuously. The volume after water into ice expands due to the action of frost heaving force, thus expands the number and width of the cracks and pores. After the ice melts into water, the water enters into the newly formed cracks. It is this repeated freeze-thaw cycle process that is the increase process of the volume and the porosity of concrete.

## 3. Impact Compression Test and Results Discussion

### 3.1. Impact Compression Test

**Figure 3:**
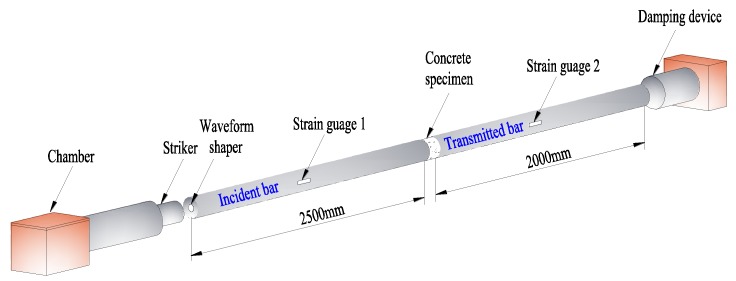
Schematic diagram of separated Hopkinson pressure bar (SHPB) device.

In this paper, directed to the concrete specimens suffered from different freeze-thaw cycles (0, 10, 20, 30, 40 and 50), the dynamic uniaxial compression tests were carried out on the Φ 50 mm separated Hopkinson pressure bar (SHPB) device (LiWei Technology Co., Ltd, Luoyang, China, shown in Figure 3) with three loading speeds (5.4 m/s, 8.8 m/s, and 11.3 m/s). To ensure the reliability of test data, the impact test was repeated three times under each condition. In order to ensure the reliability of test data, the impact compression test was repeated three times under each working condition, and a total of about 54 impact compression tests were required.

The SHPB dynamic impact test satisfies the assumption of one-dimensional stress wave and stress uniformity. According to the basic principle of the test, the pulse information is measured through the strain gauge pasted on the incident and transmission bar. The average stress, strain rate and strain of specimens are calculated by “three wave method” as shown in Formula (1)

(1) $$\left\{ \begin{array}{l} \sigma \left(t\right)=\frac{E_{0}A_{0}}{2A_{s}}\left[\varepsilon_{I}\left(t\right)+\varepsilon_{R}\left(t\right)+\varepsilon_{T}\left(t\right)\right]\\ \dot{\varepsilon }\left(t\right)=\frac{C_{0}}{L_{s}}\left[\varepsilon_{T}\left(t\right)-\varepsilon_{I}\left(t\right)-\varepsilon_{R}\left(t\right)\right]\\ \varepsilon \left(t\right)=\frac{C_{0}}{L_{s}}\int_{0}^{t}\left[\varepsilon_{T}\left(t\right)-\varepsilon_{I}\left(t\right)-\varepsilon_{R}\left(t\right)\right]dt \end{array}\right.$$

where t is a time point in the dynamic loading process; σ(t), $\dot{\varepsilon }\left(t\right)$ and ε(t) are the stress, strain rate and strain of concrete at a certain moment t, respectively; $\varepsilon_{I}\left(t\right)$, $\varepsilon_{R}\left(t\right)$ and $\varepsilon_{T}\left(t\right)$ are the incident, reflected and transmitted strain at a certain moment t, respectively; $\varepsilon_{I}\left(t\right)$ and $\varepsilon_{R}\left(t\right)$ are recorded by strain gauge 1; $\varepsilon_{T}\left(t\right)$ is recorded by strain gauge 2; $E_{0}$, $A_{0}$ and $C_{0}$ are the elastic modulus, cross-sectional area and wave velocity of the elastic bar, and take the value of $C_{0}$ as 5181 m/s herein; $A_{s}$ and $L_{s}$ are the cross-sectional area and the length of the specimen.

**Figure 4:**
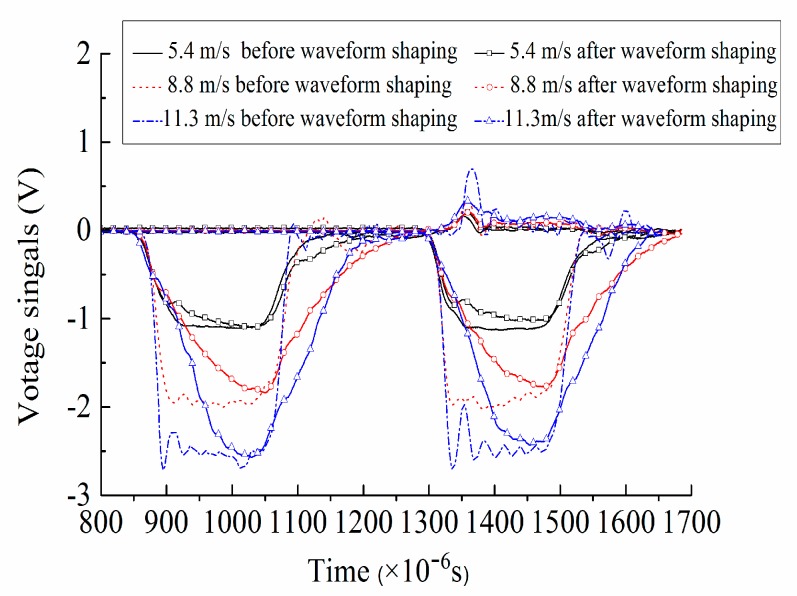
Waveform shaping images under different loading speeds.

In order to reduce the dispersion effect caused by the transverse inertial movement of bar particles and extend the time before incident pulse reaches the peak value, the waveform shaping should be considered to ensure there is sufficient time for specimens to achieve the stress uniformity before failure [22]. In this paper, a large number of tests were carried out on red copper pieces of different sizes, and the wave forms before and after shaping were compared and analyzed, just as shown in Figure 4. Finally, wave shapers suitable for different working conditions were selected, that was, when the impact loading speed was 5.4 m/s, 8.8 m/s and 11.3 m/s, the thickness of the shaper was 1 mm, and the diameter was 25 mm, 20 mm and 15 mm respectively. Before the dynamic test, the concrete specimen was sandwiched between the incident bar and transmitted bar, and the molybdenum disulfide lubricant was coated between the specimen and section of incident/transmitted bar to reduce the friction effect and the energy consumed by friction. After the pressure was loaded, the valve was manually opened and closed.

### 3.2. Test Results Discussion

**Figure 5:**
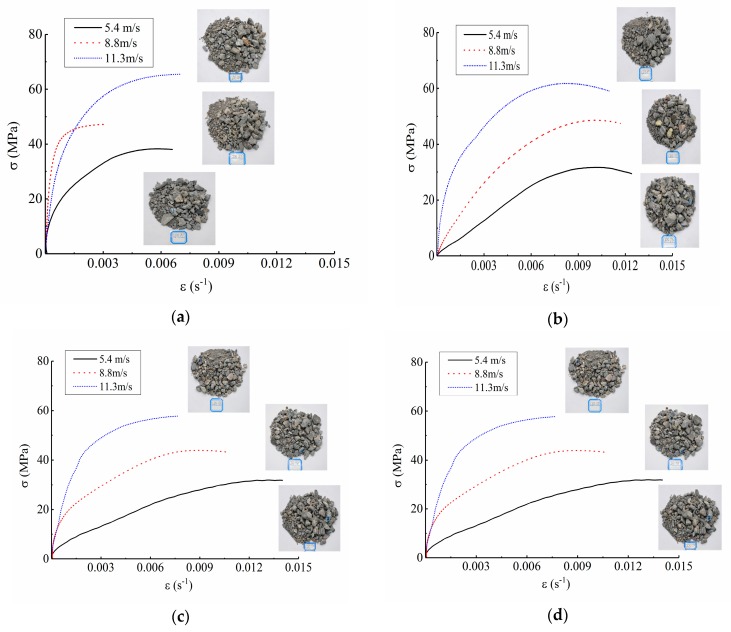

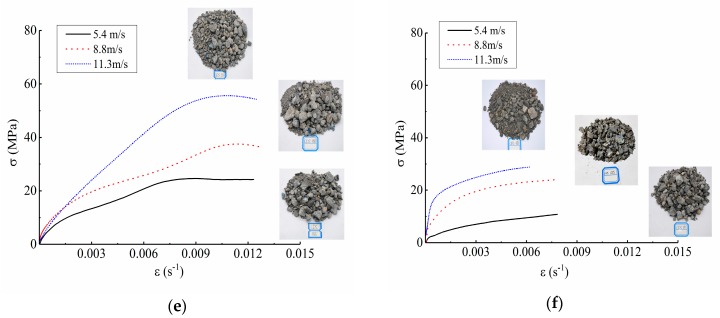
Dynamic failure modes and stress-strain curves of concrete at different impact speeds. (**a**) 0 freeze-thaw cycle; (**b**) 10 freeze-thaw cycles; (**c**) 20 freeze-thaw cycles; (**d**) 30 freeze-thaw cycles; (**e**) 40 freeze-thaw cycles; (**f**) 50 freeze-thaw cycles.

**Figure 6:**
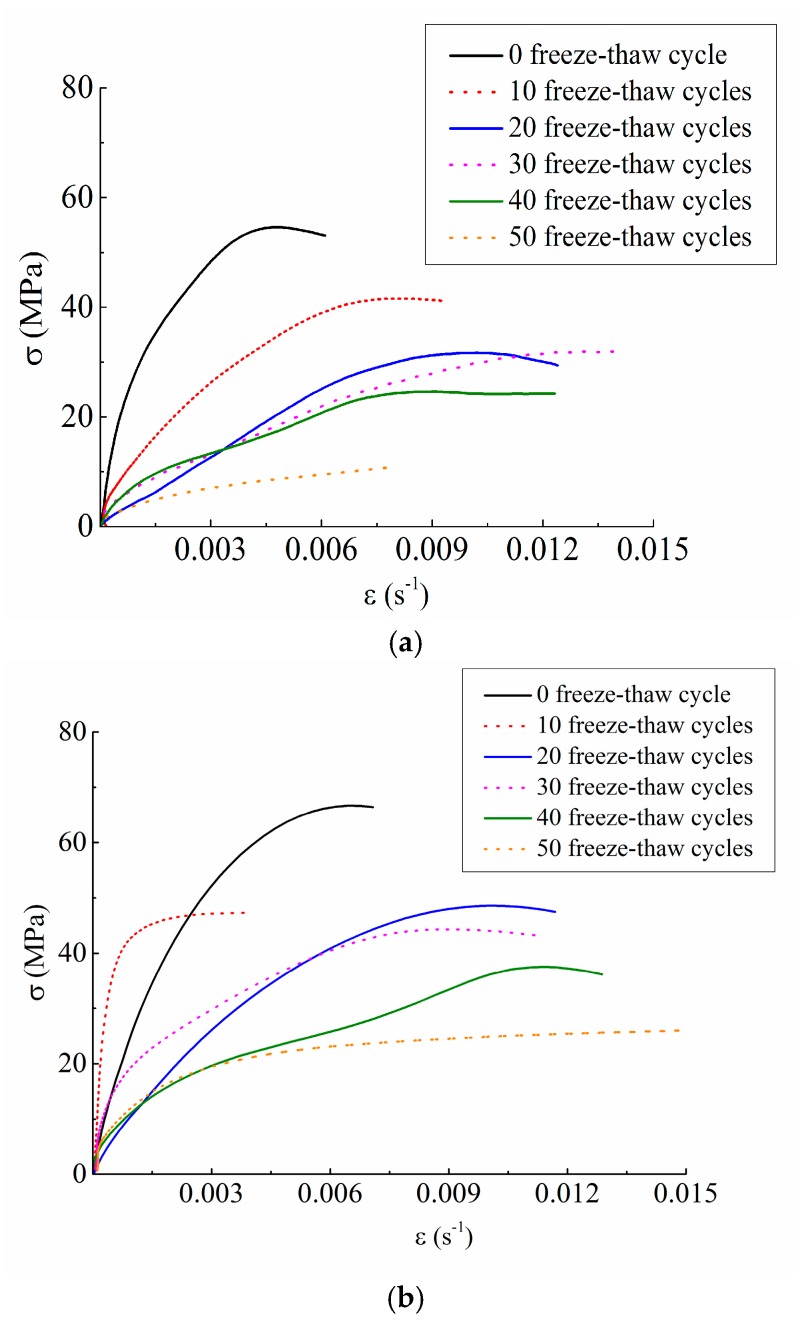

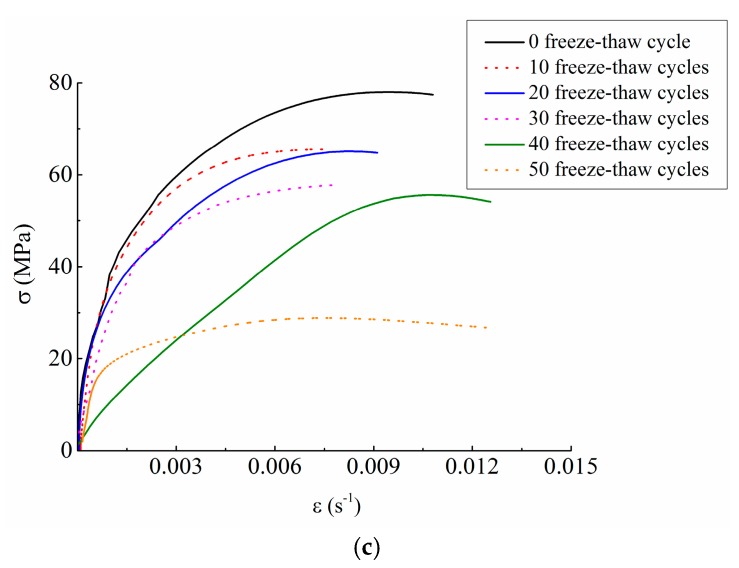
Stress-strain curves of concrete under different freeze-thaw cycle conditions. (**a**) 5.4 m/s; (**b**) 8.8 m/s; (**c**) 11.3 m/s.

Dynamic failure modes and stress-strain curves of concrete under different conditions were shown in Figure 5 and Figure 6.

The results of freeze-thaw cycle test and impact compression test indicated that, after experiencing the freeze-thaw cycle action, the internal damage defects of concrete deviated from the equilibrium state under the external impact loading. In order to dissipate the energy transferred from the outside world and reach a new balance state, cracks inside the specimen were forced to change their own structure state, continuously multiplied and gathered, and gradually developed from the disordered distribution to the orderly development, finally leading to a macroscopic catastrophic fracture. This was the impact crushing process and mechanism of concrete under freeze-thaw conditions.

As can be seen from Figure 5, the concrete showed obvious strain rate strengthening effect under any freeze-thaw cycle condition. For example, when under the condition of 0 freeze-thaw cycle, the peak stress was 55.10 MPa at the impact loading speed of 5.4 m/s, and was 68.03 MPa and 79.05 MPa at the impact loading speed of 8.8 m/s and 11.3 m/s, respectively, increasing by 23.47% and 43.47% respectively. When under the condition of 50 freeze-thaw cycles, the peak stress was 16.29 MPa at the impact loading speed of 5.4 m/s and was 27.31 MPa and 29.70 MPa at the impact loading speed of 8.8 m/s and 11.3 m/s, respectively, increasing by 67.65% and 82.32% respectively. Thus, it can be drawn that the dynamic strength of concrete has obvious rate correlation.

Figure 6 suggested that freeze-thaw cycle actions have a significant degrading effect on the mechanical properties of concrete. For instance, if under a certain loading speed of 5.4 m/s, when the freeze-thaw cycles were 0 and 50 respectively, the peak stresses were 55.10 MPa and 16.29 MPa respectively, reducing by 70.44%. If under a certain loading speed of 11.3 m/s, when the freeze-thaw cycles were 0 and 50 respectively, the peak stresses were 79.05 MPa and 29.70 MPa respectively, reducing by 62.43%. Based on the above analysis, we can draw that the dynamic mechanical properties of concrete in a particular freeze-thaw environment are the result of the coupling effect of strain rate strengthening effect and freeze-thaw deterioration effect.

In addition, it was also shown in Figure 5 that when the freeze-thaw cycles were less or the loading speed was lower, the dynamic compression failure patterns of concrete specimens were strips and block fragments with large and medium grain size, such as the condition of 0 freeze-thaw cycle with loading speed of 5.4 m/s. As the number of freeze-thaw cycles increased or the loading speed improved, the crushing degree of concrete specimens became more and more serious, and most of them were uniform fine granular powder, such as the condition of 50 freeze-thaw cycles with loading speed of 11.3 m/s. The reason for this phenomenon was as follows: when the freeze-thaw cycles were less or the loading speed was lower, there were fewer internal cracks in the specimen, which directly extended and ran through in situ under the dynamic load, leading to a small degree of the fracture and a splitting failure mode. However, when the freeze-thaw cycles were more or the loading speed was higher, the axial and transverse cracks of specimens increased, which propagated and penetrated unstably under the dynamic load, and the specimens were “cut” into small particles rapidly, resulting in a severe crushing failure and a crushing failure mode.

**Figure 7:**
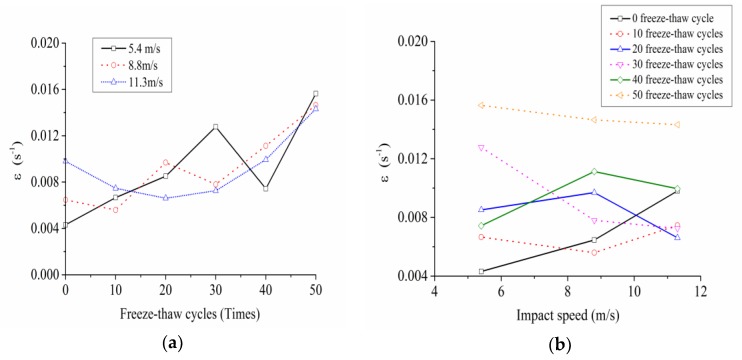
Variation rules of dynamic peak strain with freeze-thaw cycles and impact loading speed. (**a**) Variation rules of dynamic peak strain with freeze-thaw cycles; (**b**) variation rules of dynamic peak strain with impact loading speed.

The variation rules of dynamic peak strain with freeze-thaw cycles and impact loading speed were shown in the Figure 7.

As can be seen from Figure 7, the dynamic peak strain had no obvious dependence on the freeze-thaw cycle actions and impact loading speeds.

## 4. Fractal Characteristics of Crushing Blocks

### 4.1. Screening Statistical Test

**Figure 8:**
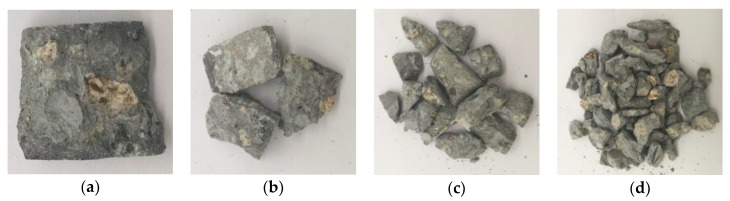

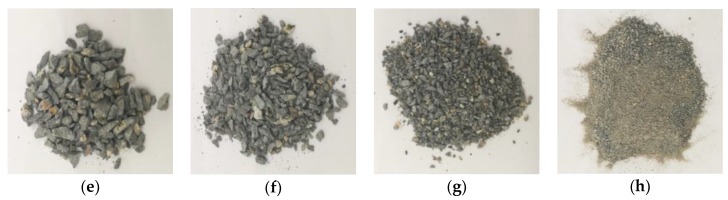
Particle size distribution of crushing blocks under the condition of 0 freeze-thaw cycle with loading speed of 5.4 m/s. (**a**) Greater than 19 mm; (**b**) 16~19 mm; (**c**) 9.6~16 mm; (**d**) 4.75~9.6 mm; (**e**) 2.36~4.75 mm; (**f**) 1~2.36 mm; (**g**) 0.5~1 mm; (**h**) Smaller than 0.5 mm.

The concrete crushing blocks were collected after the impact test and the screening statistical test was conducted on the ZBSX-92a standard vibrator (Xingye Test Instrument Co., Ltd., Cangzhou, China). The diameter of the sieve holes are 0.5 mm, 1 mm, 2.36 mm, 4.75 mm, 9.6 mm, 16 mm and 19 mm respectively. The pendulum instrument shakes 221 times and vibrates 147 times per minute, with the swing of 25 mm. The motor is 1400 revolutions per minute. Fragments of all particle sizes can be effectively separated through the sieve. Taking the condition of 0 freeze-thaw cycle with loading speed of 5.4 m/s as an example, the crushing blocks of different particle sizes were shown in Figure 8. After the screening test, weighed the mass of objects under each stage with high sensitivity electronic scale and recorded the test data.

### 4.2. Scale-Mass Distribution Rules of Crushing Blocks

**Figure 9:**
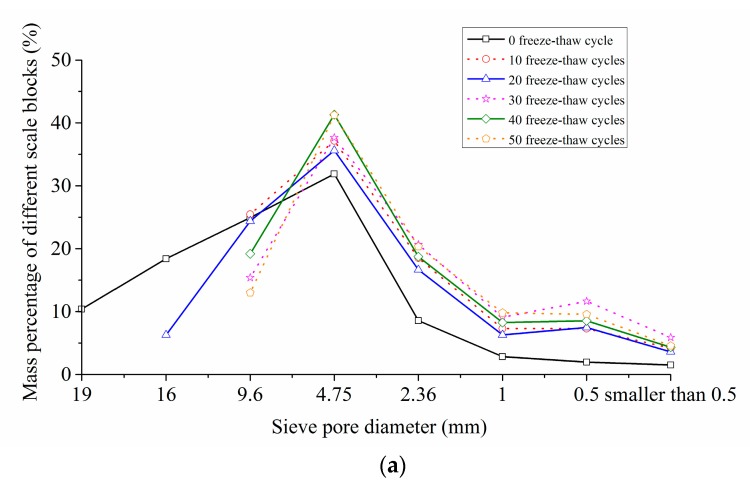

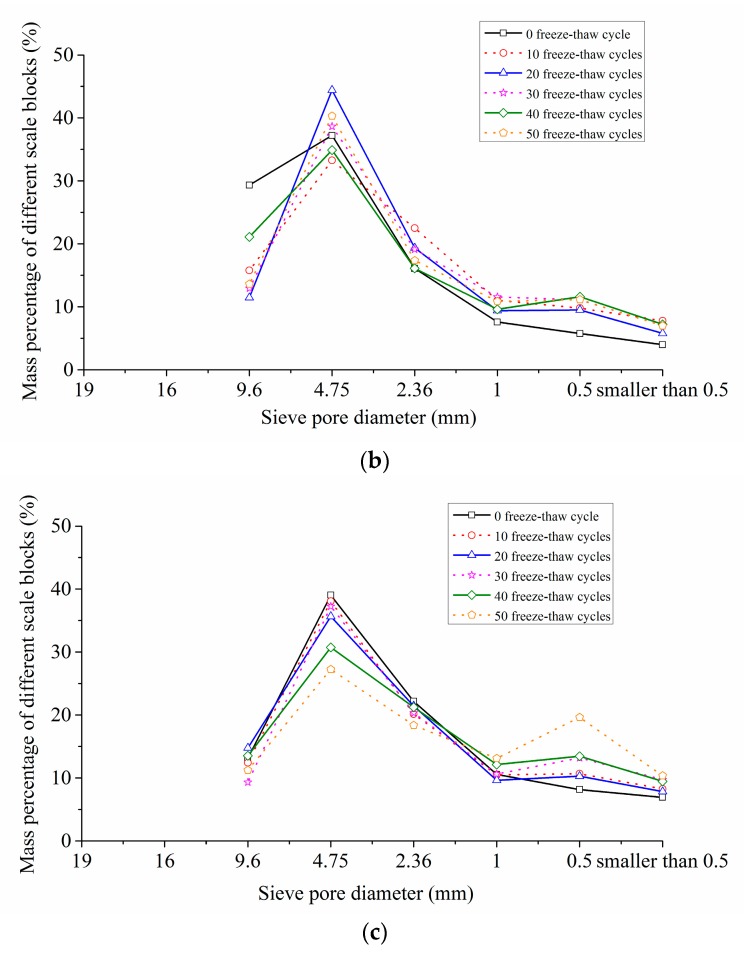
Scale-mass distribution curves of impact fragments. (**a**) 5.4 m/s; (**b**) 8.8 m/s; (**c**) 11.3 m/s.

The screening test results of concrete crushing blocks under different freeze-thaw cycle conditions and loading speeds were shown in Table 5. The scale-mass distribution curves of impact fragments under different conditions were shown in Figure 9.

It can be seen from Table 5 and Figure 9 that, under the various working conditions, the crushing blocks of particle size between 4.75 mm and 9.6 mm accounted for the largest percentage, about 27.26~44.41%. Among them, blocks of particle size over 19 mm and particle size of 16~19 mm only appeared in the condition of 0 freeze-thaw cycle with impact loading speed of 5.4m/s, and the mass percentage was 10.39% and 18.40% respectively. With the improvement of the impact loading speed, the mass percentage of the large particle size blocks decreased gradually, and that of the small particle size blocks increased accordingly. For example, under the condition of 0 freeze-thaw cycle, when the impact loading speeds were 5.4 m/s, 8.8 m/s and 11.3 m/s, the particle mass percentages greater than or equal to 9.6 mm were 53.73%, 29.32% and 13.14%, respectively, and those less than or equal to 4.75 mm were 46.75%, 70.64% and 86.84%, respectively. This phenomenon demonstrated that the greater the impact loading speed was, the more serious the specimen was broken.

It also can be found from Table 5 and Figure 9 that, when at a certain loading rate, with the increase of the freeze-thaw cycles, the mass percentage of large particle size blocks decreased gradually, and that of small particle size blocks increased consequently. For instance, when the loading speed was 5.4 m/s, and the freeze-thaw cycles were 0 and 50 respectively, the particle mass percentages greater than or equal to 9.6 mm were 53.73% and 13.00% respectively, and those less than or equal to 4.75 mm were 46.75% and 85.56% respectively. This meant that the more freeze-thaw cycles the concrete experienced, the more serious the concrete damage was.

In conclusion, due to the effect of the freeze-thaw degradation and the impact energy, the freeze-thaw action and loading speed had a significant correlation with the scale-mass distribution rules of crushing blocks. Moreover, the more freeze-thaw cycles the concrete suffered, or the higher the loading speed was, the more serious the crushing degree of specimens would be, and the larger the mass proportion of the small and medium size fragments would be. The mechanism of this phenomenon was as follows: when freeze-thaw cycles are less or the loading speed is lower, only those microscopic cracks that consume less energy during the expansion have practical effects on the impact crushing of concrete. The propagation and penetration of these microscopic cracks will lead to concrete fracture failure before the absorption energy increases enough to cause other microscopic cracks to crack and form the main crack. When more freeze-thaw cycles or a higher loading speed is experienced, the energy absorbed by the concrete reaches a higher level before the mesoscopic crack is penetrated, so that more mesoscopic cracks can expand and participate in the crushing process, leading to a smaller size of specimen broken blocks.

### 4.3. Fractal Characteristics of Crushing Blocks

According to the mass-frequency relationship [23], the distribution equation of concrete fragments under the impact loading is

(2) $$Y=\frac{M_{r}}{M_{T}}={\left(\frac{r}{r_{m}}\right)}^{3-D}$$

where r, $r_{m}$ and D are the particle size, the maximum size and the fractal dimension of crushing blocks respectively; $M_{r}$ is the total mass of crushing blocks with particle size smaller than r; $M_{T}$ is the total mass of specimen crushing blocks.

Take the natural logarithm on the both sides of Equation (2) to get

(3) $$\ln Y=\ln\left(\frac{M_{r}}{M_{T}}\right)=\left(3-D\right)\ln\left(\frac{r}{r_{m}}\right)$$

In the ln*r*–ln *(M_r_/M_T_)* coordinate system, the slope of the fitting line is K=3−D. Therefore, the fractal dimension of crushing blocks can be calculated by the mass-granularity method [24] to be

(4) D=3−K

**Figure 10:**
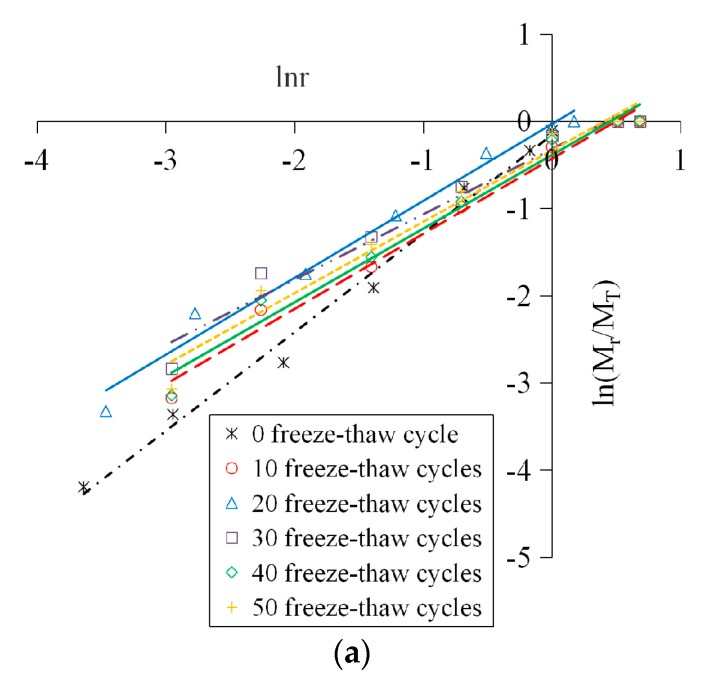

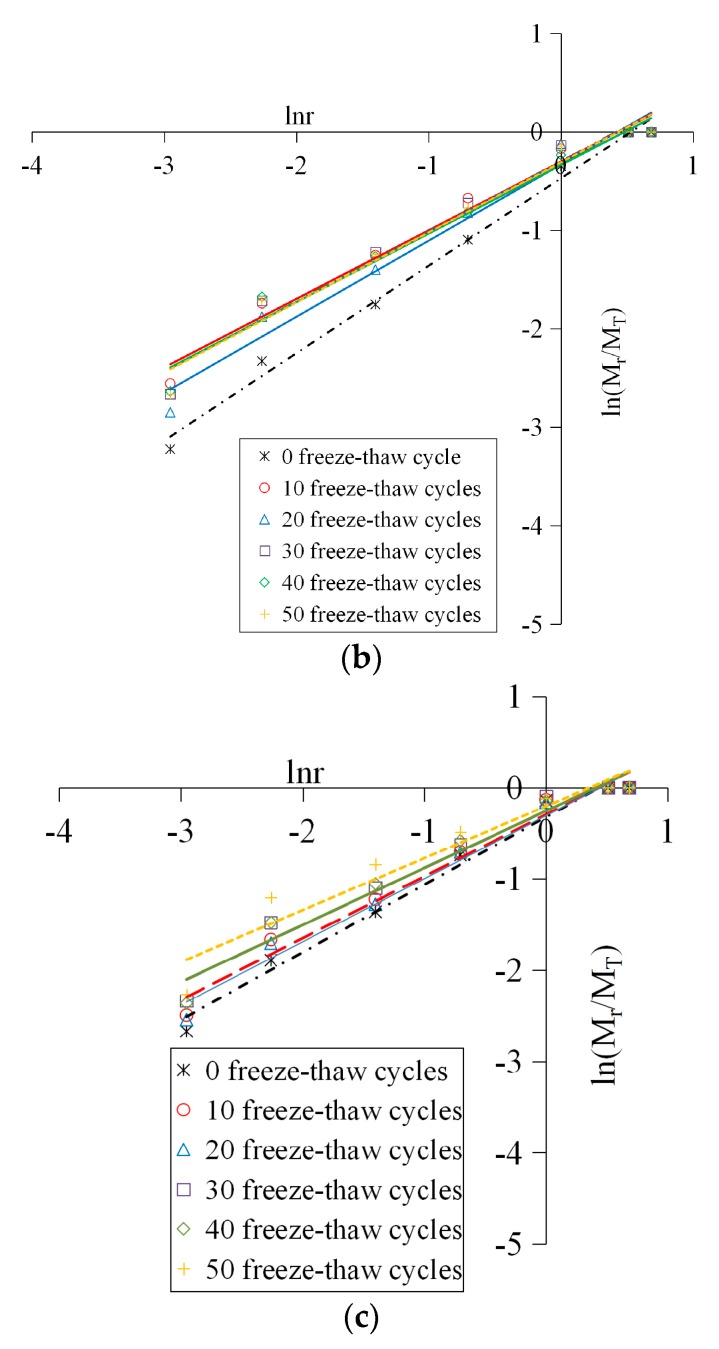
Logarithmic curves of concrete crushing blocks. (**a**) 5.4 m/s; (**b**) 8.8 m/s; (**c**) 11.3 m/s.

The logarithmic curves of concrete crushing blocks under different freeze-thaw cycles and loading speeds were shown in Figure 10.

It can be found in Figure 10 that all data points under different working conditions showed a good linear correlation in the ln*r*–ln *(M_r_/M_T_)* coordinate system, indicating that the distribution of the concrete fragments had fractal characteristics. This is because the distribution of the mesoscopic cracks and pores inside the concrete conforms to the fractal theory, and has self-similarity under different scales. The freeze-thaw deterioration and the impact crushing process are the direct results of crack extension, thus the crushing blocks of specimens suffered different freeze-thaw cycles and impact loadings also show a certain self-similarity, that is, they satisfy the power law characteristics and it is a fractal of statistical significance. In summary, the concrete material has fractal properties from the microscopic damage to the macroscopic fracture, and the larger the fractal dimension is, the more fragments there are, the smaller the fragment size is, and the more serious the material breakage is.

**Figure 11:**
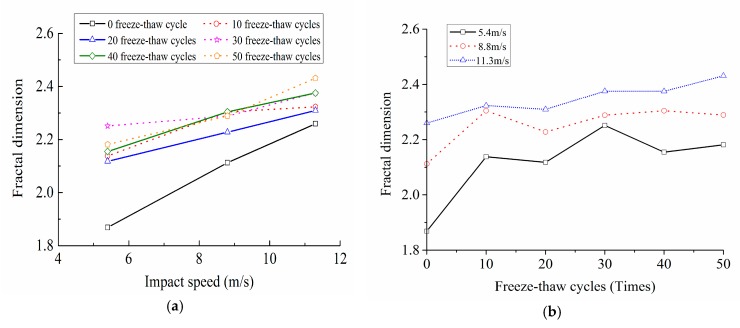
Variation rules of fractal dimension with loading speed and freeze-thaw cycles. (**a**) Variation rules of fractal dimension with loading speed; (**b**) Variation rules of fractal dimension with freeze-thaw cycles.

**Figure 12:**
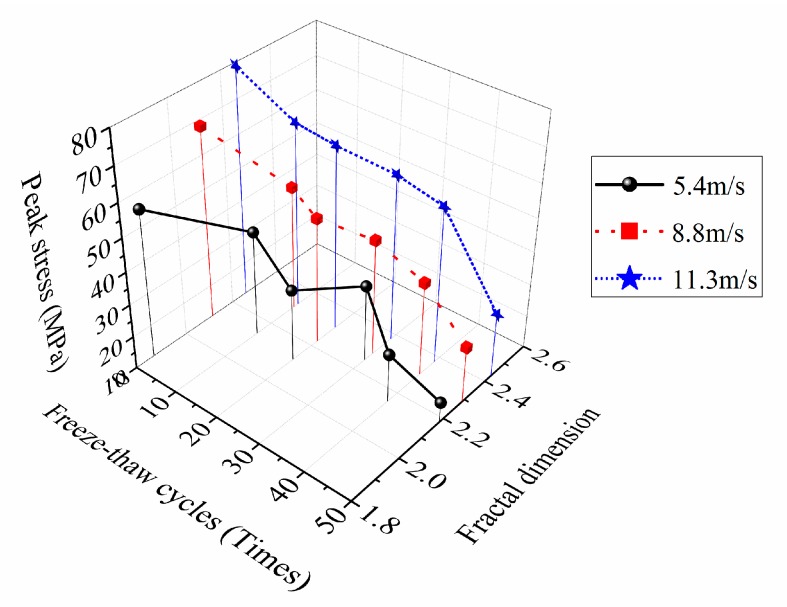
Relationship curves between the fractal dimension and the dynamic peak stress of concrete.

The variation rules of fractal dimension with loading speed and freeze-thaw cycles were shown in Figure 11. The relationship curves between the fractal dimension and the dynamic peak stress of concrete were shown in Figure 12.

Figure 11 indicated that the fractal dimension significantly increased with the increase of the loading speed, namely, the larger the loading speed was, the higher the broken degree of the concrete was. In the range of 0 to 50 freeze-thaw cycles, the fractal dimensions of concrete at the loading speeds of 5.4 m/s, 8.8 m/s and 11.3 m/s were 1.87~2.18, 2.11~2.29 and 2.26~2.43 respectively.

Figure 11 also showed that, when the loading speed changed from 5.4 m/s to 11.3 m/s, the fractal dimensions of concrete under six kind of freeze-thaw cycles were 1.87~2.26, 2.14~2.32, 2.12~2.31, 2.25~2.38, 2.15~2.38 and 2.18~2.43, respectively. It can be seen that, when at the same loading speed, the fractal dimension generally rose with the growth of the freeze-thaw cycles, and its increment reached the maximum under the 0 freeze-thaw cycle condition. This is because as the growth of the freeze-thaw cycles, the freeze-thaw damage intensifies, the cohesion at the interface of each phase inside the concrete decreases, and the micro-cracks increase; the performance of the specimen seriously deteriorates, and the fractal dimension of impact broken blocks increases therefore.

Figure 12 suggested that, when under a certain loading rate, the more freeze-thaw cycles the concrete suffered, the higher the damage degree was, the worse the mechanical property was, the lager the fractal dimension of crushing blocks was, and the smaller the dynamic peak stress was. In addition, Figure 12 also illustrated that, when under the same freeze-thaw cycle condition, although the fractal dimension increased with the increase of the loading speed due to the obvious rate correlation of the concrete dynamic strength, the dynamic peak stress tended to increase, nevertheless. Thus we can draw a conclusion that the distribution rules of concrete dynamic peak stress varying with the fractal dimension is coupling affected by the freeze-thaw cycles and the loading speed, and this is a coupling mechanism in which freeze-thaw deterioration effect and strain rate strengthening effect coexist and compete.

## 5. Analysis of Energy Dissipation

According to the thermodynamic theorem, the energy transformation is the essential characteristic of physical change processes of the matter, while the material destruction is the instability phenomenon driven by the energy [2]. The energy consumption characteristics of concrete under freeze-thaw cycle actions mainly depend on two factors [4]: the initial damage state inside the concrete and the external energy acting on the material. The former is mainly affected by the number of freeze-thaw cycles. The microscopic damage degree of specimens under different freeze-thaw cycle conditions is various, leading to diversities in their ability to resist stress waves, and thus affecting the macroscopic energy absorption characteristics of specimens. The latter is mainly affected by the loading rate. Each stage of the crack initiation, propagation, expansion, aggregation and penetration, is a process of irreversible energy consumption.

### 5.1. Energy Dissipation Principles of SHPB Test

According to the one-dimensional stress wave theory and the energy conservation law, the calculation formula of incident energy, reflected energy and transmitted energy of SHPB test is [25]

(5) $$\left\{ \begin{array}{l} W_{I}=\frac{A_{0}C_{0}}{E_{0}}\int \sigma_{I}^{2}dt=A_{0}C_{0}E_{0}\int \varepsilon_{I}^{2}dt\\ W_{R}=\frac{A_{0}C_{0}}{E_{0}}\int \sigma_{R}^{2}dt=A_{0}C_{0}E_{0}\int \varepsilon_{R}^{2}dt\\ W_{T}=\frac{A_{0}C_{0}}{E_{0}}\int \sigma_{T}^{2}dt=A_{0}C_{0}E_{0}\int \varepsilon_{T}^{2}dt \end{array}\right.$$

where $W_{I}$, $W_{R}$ and $W_{T}$ are the incident energy, reflected energy and transmitted energy respectively. $\sigma_{I}$, $\sigma_{R}$ and $\sigma_{T}$ are the stress time history of the incident wave, reflected wave and transmitted wave respectively. $\varepsilon_{I}$, $\varepsilon_{R}$ and $\varepsilon_{T}$ are the strain time history of the incident wave, reflected wave and transmitted wave respectively.

Ignoring the energy loss caused by the friction between the specimen and section of incident/transmitted bar, then the total energy absorbed by the concrete specimen during the impact crushing process is

(6) $$W=W_{I}-(W_{R}+W_{T})$$

where W is the total energy absorbed by the concrete specimen during the impact crushing process, which is mainly divided into four parts [26]: fracture energy $W_{F}$, mainly used for the formation of the fracture surface; damage energy $W_{D}$, mainly used for the crack expansion, the microcrack propagation and so on; kinetic energy $W_{K}$ of broken blocks; other forms of dissipated energy $W_{O}$, such as thermal energy, radiant energy, etc. Among them, both $W_{D}$ and $W_{O}$ are very small, which can be ignored. Then, the total absorption energy W can be written as

(7) $$W=W_{F}+W_{K}$$

According to the research result of literature [27], the relationship between the kinetic energy $W_{K}$ and the total absorption energy W in the SHPB test can be expressed as

(8) $$\frac{W_{K}}{W}=\frac{0.69v+0.22}{100}$$

where v is the impact loading speed, m/s.

In order to eliminate the influence of the specimen size, the energy consumption density, namely the absorbed energy per unit volume of the specimen, was introduced to reflect the energy consumption characteristics of concrete materials

(9) $$\zeta =\frac{W}{V}$$

where ζ and V were the energy consumption density and the volume of concrete specimens respectively.

### 5.2. Analysis on Calculation Results of the Energy Consumption

**Figure 13:**
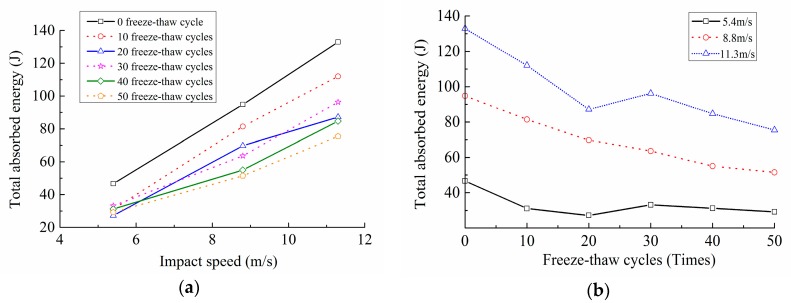
Relation curves of the total absorption energy with freeze-thaw cycles and impact loading speeds. (**a**) Relation curves of the total absorption energy with impact loading speeds; (**b**) Relation curves of the total absorption energy with freeze-thaw cycles.

The results calculated by Equations (6)–(9), including the incident energy $W_{I}$, total absorption energy W, fracture energy $W_{F}$, block kinetic energy $W_{K}$ and the energy consumption density ζ, were shown in Table 6. The relation curves of the total absorption energy W with the freeze-thaw cycles and the impact loading speed were shown in Figure 13.

As shown in Table 6, there was about 92~96% of the total absorbed energy converted into fracture energy for the formation of the new fracture surface, and there was only about 4~8% converted into the kinetic energy of broken blocks. Furthermore, with the increase of the impact loading speed, the ratio of the absorption energy converted into fracture energy decreased gradually, and the ratio of the absorption energy converted into kinetic energy increased accordingly.

Both Table 6 and Figure 13 indicated that, when the number of the freeze-thaw cycle was constant, the higher the impact loading speed was, the greater the incident energy was, and the more energy the concrete specimens absorbed during the impact crushing process. Under this condition, there was no time for the internal cracks to propagate and penetrate along the weakest interface. Instead, a large number of new micro-cracks were generated at the same time in their respective regions to consume the external impact energy. Therefore, the number of the micro-cracks inside the specimen increased, the degree of the impact crushing was severe, and the size of the broken blocks was smaller, which was consistent with the failure pattern shown in Figure 5. Moreover, when the impact loading speed was constant, the more freeze-thaw cycles the concrete experienced, the less energy the specimen absorbed. This was because the energy consumed by the crack generation was far higher than that by the crack propagation [4]. With the increase of the number of freeze-thaw cycles, the primary cracks inside concrete increased, the initial deterioration degree developed, and the integrity and compactness of concrete specimens were damaged consequently. When the impact loading was applied, the internal cracks accelerated to expand, and the specimens were destroyed sharply, making the absorption energy decreased.

### 5.3. Relationship between the Energy Consumption Density and the Fractal Dimension

The difference in the dissipation of the external impact energy and that in the failure patterns of crushing blocks, are the essential internal cause and the macroscopic representation respectively of the concrete crushing characteristics under different freeze-thaw cycle conditions and impact loading speeds [4,12]. Therefore, there must be an intrinsic relationship between the dissipated energy and the fractal dimension of fragments in the concrete impact crushing process.

**Figure 14:**
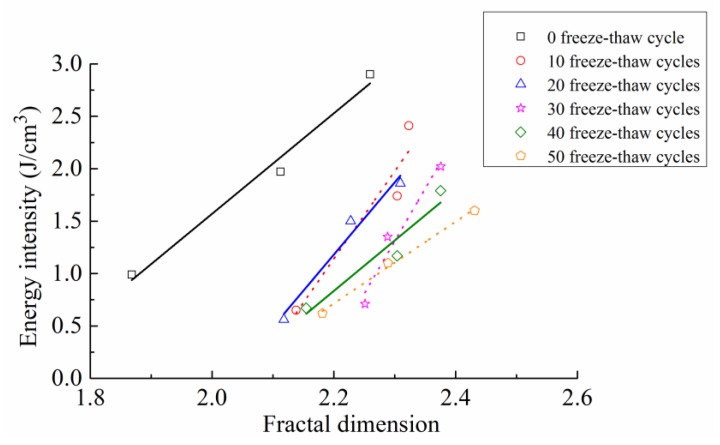
Relation fitting curves of the energy consumption density and the fractal dimension.

The relation fitting curves of the energy consumption density and the fractal dimension of concrete specimens under different conditions were shown in Figure 14, and the fitting formula expressions and correlation coefficients were shown in Table 7.

As can be found in Figure 14, when the number of freeze-thaw cycles was constant, the energy consumption density of concrete under three different impact loading speeds increased with the increase of the fractal dimension, suggesting that the more energy the specimen absorbed, the more developed the crack was, and the more thorough the specimen destruction was. In addition, when the impact loading speed was constant, with the growth of the freeze-thaw cycles, the energy consumption density reduced under a same fractal dimension condition, while the fractal dimension improved under a same energy consumption density condition, indicating that freeze-thaw cycle actions had obvious damage and deterioration effects on the concrete materials.

Table 7 summarized the formulas and parameters of the energy consumption density under different freeze-thaw cycle conditions. The correlation coefficients resulted from the six freeze-thaw cycle conditions were 0.9919, 0.9069, 0.9716, 0.9528, 0.9294 and 0.9976, respectively. This illustrated that there was a significant positive linear correlation between the energy consumption density and the fractal dimension of concrete suffered freeze-thaw cycle actions, and the relationship could be expressed as ξ=aD+b, where a and b were the fitting coefficients.

## 6. Conclusions

(1) The impact crushing mechanism and process of concrete under freeze-thaw conditions are as follows: after experiencing the freeze-thaw cycle action, the internal damage defects of concrete deviate from the equilibrium state under the external impact loading. In order to dissipate the energy transferred from the outside world and reach a new balance state, cracks inside the specimen are forced to change their own structure state, continuously multiply and gather, and gradually develop from the disordered distribution to the orderly development, finally leading to a macroscopic catastrophic fracture.

(2) The freeze-thaw cycle action and the impact loading speed have a significant effect on the scale-mass distribution rules of crushing blocks, and the more freeze-thaw cycles the concrete suffered, or the higher the loading speed is, the more serious the crushing degree of specimens will be, and the larger the mass proportion of the small and medium size fragments will be.

(3) The concrete material has fractal properties from the microscopic damage to the macroscopic fracture. The fractal dimension increases significantly with the increase of the impact loading speeds and improves generally with the growth of the number of freeze-thaw cycles. The increment of the fractal dimension reaches the maximum under the 0 freeze-thaw cycle condition. The distribution rules of concrete dynamic peak stress varying with fractal dimension is coupling affected by the freeze-thaw cycles and the loading speeds.

(4) The impact fracture of concrete under different freeze-thaw cycle conditions and loading speeds is a fractal evolution process driven by the external impact energy. The energy dissipation is intrinsically related to the fractal characteristics, and the energy consumption density increases with the increase of the fractal dimension under a certain freeze-thaw cycle condition. When at a certain loading speed, with the growth of the freeze-thaw cycles, the energy consumption density reduces under the same fractal dimension, while the fractal dimension improves under the same energy consumption density.

## Figures and Tables

**Table 1:** Mixing proportions of concrete specimens.

Item	Cement	Gravel	Sand	Fly Ash	Water Reducing Agent	Water
Proportion	1	3.80	2.03	0.40	0.02	0.56

**Table 2:** Physical and chemical properties of cement.

Item	Density g/cm^3^	Initial Setting Time (min)	Final Setting Time (min)	Stability	Flexural Strength (MPa)	Compressive Strength (MPa)
Values	3.05	208	260	qualified	8.6	51

**Table 3:** Gravel grading.

**Sieve Diameter (mm)**	19.0	16.0	9.5	4.75	2.36
**Cumulative weight of screen residue (%)**	0	2	42	97	99

**Table 4:** Sand grading.

**Sieve Diameter (mm)**	4.75	2.36	1.25	0.63	0.315	0.16
**Cumulative weight of screen residue (%)**	2.8	9.7	21.4	48.1	70.5	99.4

**Table 5:** Mass percentage of each particle size under different conditions.

Impact Loading Speed (m/s)	Freeze-Thaw Cycles (Times)	Greater Than 19 mm	16~19 mm	9.6~16 mm	4.75~9.6 mm	2.36~4.75 mm	1~2.36 mm	0.5~1 mm	Smaller Than 0.5 mm
5.4	0	10.39	18.40	24.94	31.88	8.56	2.83	1.96	1.52
	10	—	—	25.47	37.16	18.52	7.30	7.30	4.17
	20	—	6.28	24.39	35.63	16.65	6.30	7.48	3.61
	30	—	—	15.40	37.65	20.67	9.11	11.65	5.89
	40	—	—	19.19	41.32	18.74	8.27	8.54	4.32
	50	—	—	13.00	41.27	20.33	9.81	9.57	4.58
8.8	0	—	—	29.32	37.22	16.09	7.58	5.76	3.99
	10	—	—	15.78	33.26	22.51	11.04	9.80	7.78
	20	—	—	11.46	44.41	19.36	9.40	9.50	5.79
	30	—	—	12.95	38.66	19.13	11.57	11.11	7.01
	40	—	—	21.09	34.89	16.11	9.60	11.62	7.21
	50	—	—	13.63	40.30	17.37	10.87	11.15	7.00
11.3	0	—	—	13.14	39.03	22.17	10.56	8.16	6.92
	10	—	—	12.43	38.08	20.16	10.50	10.67	8.27
	20	—	—	14.78	35.66	21.45	9.64	10.28	7.84
	30	—	—	9.31	37.26	20.33	10.62	13.20	9.73
	40	—	—	13.53	30.74	21.24	12.14	13.46	9.47
	50	—	—	11.23	27.26	18.37	13.12	19.63	10.35

**Table 6:** Calculation results of the energy consumption under different conditions.

No.	Specimen Number	Freeze-Thaw Cycles (Times)	v (m/s)	$W_{I}\;(J)$	W (J)	$W_{F}\;(J)$	$W_{K}\;(J)$	$W_{F}/W\;(\%)$	$W_{K}/W\;(\%)$	$\zeta \;(J/{\mathrm{cm}}^{3})$
1	CDS3	0	5.4	97.45	46.65	44.81	1.84	96.05	3.95	0.99
2	CDS4	0	8.8	339.34	94.83	88.86	5.97	93.71	6.29	1.97
3	CDS7	0	11.3	502.28	132.90	122.25	10.65	91.98	8.02	2.90
4	CDS37	10	5.4	90.01	31.08	29.85	1.23	96.05	3.95	0.65
5	CDS41	10	8.8	292.17	81.44	76.32	5.12	93.71	6.29	1.74
6	CDS44	10	11.3	474.74	111.99	103.01	8.98	91.98	8.02	2.41
7	CDS73	20	5.4	84.08	27.20	23.13	1.07	96.05	3.95	0.56
8	CDS77	20	8.8	276.27	69.74	65.35	4.39	93.71	6.29	1.50
9	CDS80	20	11.3	548.70	87.21	80.22	6.99	91.98	8.02	1.86
10	CDS110	30	5.4	99.04	33.20	31.89	1.31	96.05	3.95	0.71
11	CDS112	30	8.8	287.92	63.59	59.59	4.00	93.71	6.29	1.35
12	CDS115	30	11.3	451.84	96.23	88.52	7.71	91.98	8.02	2.02
13	CDS145	40	5.4	89.96	31.20	29.97	1.23	96.05	3.95	0.67
14	CDS148	40	8.8	281.76	54.98	51.52	3.46	93.71	6.29	1.17
15	CDS152	40	11.3	466.64	84.72	77.93	6.79	91.98	8.02	1.79
16	CDS181	50	5.4	105.08	29.20	28.05	1.15	96.05	3.95	0.62
17	CDS185	50	8.8	357.04	51.48	48.24	3.24	93.71	6.29	1.10
18	CDS188	50	11.3	523.69	75.46	69.41	6.05	91.98	8.02	1.60

**Table 7:** Fitting formula expressions and correlation coefficients.

Freeze-Thaw Cycles (Times)	Fitting Formula Expressions	R	R^2^
0	ξ=4.7947D−8.0205	0.9919	0.9839
10	ξ=8.3529D−17.236	0.9069	0.8224
20	ξ=6.8941D−13.986	0.9716	0.9440
30	ξ=10.024D−21.746	0.9528	0.9079
40	ξ=4.8126D−9.7537	0.9294	0.8638
50	ξ=3.9002D−7.8652	0.9976	0.9953
